# BTK Promotes Atherosclerosis by Regulating Oxidative Stress, Mitochondrial Injury, and ER Stress of Macrophages

**DOI:** 10.1155/2021/9972413

**Published:** 2021-05-27

**Authors:** Junxiong Qiu, Yuan Fu, Zhiteng Chen, Lisui Zhang, Ling Li, Diefei Liang, Feng Wei, Zhuzhi Wen, Yajing Wang, Shi Liang

**Affiliations:** ^1^Department of Cardiovascular Surgery, Sun Yat-sen Memorial Hospital, Sun Yat-sen University, Guangzhou, China 510120; ^2^Department of Cardiology, Sun Yat-sen Memorial Hospital, Sun Yat-sen University, Guangzhou, China 510120; ^3^Department of Endocrinology, Sun Yat-sen Memorial Hospital, Sun Yat-sen University, Guangzhou, China 510120; ^4^Department of Otorhinolaryngology-Head and Neck Surgery, Sun Yat-sen Memorial Hospital, Sun Yat-sen University, Guangzhou, China 510120

## Abstract

Atherosclerosis (AS) is a chronic metabolic disease in arterial walls, characterized by lipid deposition and persistent aseptic inflammation. AS is regarded as the basis of a variety of cardiovascular and cerebrovascular diseases. It is widely acknowledged that macrophages would become foam cells after internalizing lipoprotein particles, which is an initial factor in atherogenesis. Here, we showed the influences of Bruton's tyrosine kinase (BTK) in macrophage-mediated AS and how BTK regulates the inflammatory responses of macrophages in AS. Our bioinformatic results suggested that BTK was a potential hub gene, which is closely related to oxidative stress, ER stress, and inflammation in macrophage-induced AS. Moreover, we found that BTK knockdown could restrain ox-LDL-induced NK-*κ*B signaling activation in macrophages and repressed M1 polarization. The mechanistic studies revealed that oxidative stress, mitochondrial injury, and ER stress in macrophages were also suppressed by BTK knockdown. Furthermore, we found that sh-BTK adenovirus injection could alleviate the severity of AS in ApoE^−/−^ mice induced by a high-fat diet in vivo. Our study suggested that BTK promoted ox-LDL-induced ER stress, oxidative stress, and inflammatory responses in macrophages, and it may be a potential therapeutic target in AS.

## 1. Introduction

AS, which underlies myocardial infarction, vascular occlusive disease, and stroke, is a chronic metabolic disease in arterial walls, characterized by lipid deposition and persistent aseptic inflammation [1]. AS is an important component in the pathobiology of many cardiovascular diseases, involving vascular endothelial activation, monocyte recruitment, and cholesterol accumulation in macrophages [2]. It is widely recognized that macrophages, which would become foam cells after internalizing lipoprotein particles, play an important role in atherogenesis [3–5]. Oxidative low-density lipoprotein (ox-LDL) is contributing to atherogenesis, foam cell formation, and AS progression [6]. M1 macrophages, with the low ability to clear cholesterol and high susceptibility to become foam cells, are involved in the initial progression of AS by producing a variety of proinflammatory factors, as well as associated with endoplasmic reticulum stress (ER stress) and mitochondrial stress [7–9]. Excessive reactive oxygen species (ROS), produced by ER, mitochondria, and other organelles, participated not only in the initial development of atherogenesis but also in many cardiovascular diseases [10]. Mitochondria-associated membranes (MAMs) are a molecule dynamic platform that is tightly associated with ER and mitochondria, and MAMs regulated multiple signaling pathways, especially inflammatory pathways, which may have an impact on the responses of macrophages [11]. In the meantime, M2 macrophages, with anti-inflammatory factor secretion and high phagocytic ability, would subsequently shift to the M1 type at the advanced stage of AS [12, 13].

BTK is a member of the Tec family in nonreceptor tyrosine kinases that regulate innumerable processes, such as cell development, proliferation, differentiation, innate immunity, and adaptative immunity [14]. Given its established roles in immune B cell inflammation, some studies reported that BTK is a drug target for B cell malignancies and autoimmune disorders [15, 16]. Functionally, BTK is considered to activate the NF-*κ*B signaling pathway by phosphorylating I*κ*B*α* protein, a main repressor of NF-*κ*B, as well as promoting the nuclear translocation of p65, and some researchers found that suppressing BTK would block the B cell receptor-dependent NF-*κ*B signaling pathway [17, 18]. NF-*κ*B signaling is regarded as a significant pathway related to inflammation in atherosclerotic plaque formation, and the genes that target the NF-*κ*B signaling pathway may provide an antiatherogenic profile [19]. Accumulating evidence revealed that BTK inhibition could make sense on tumor metastasis and infection by regulating macrophage polarization, phagocytosis, and proinflammatory factor secretion, which suggested a viable therapeutic opportunity in inflammatory diseases, such as AS [20–23]. Importantly, it is reported that BTK inhibitors, such as acalabrutinib and ONO/GS-4059, made a difference in AS treatment as antiplatelet drugs to inhibit platelet aggregation in atherosclerotic plaques [24]. However, the role of BTK in macrophages on the progression of AS has not been elucidated, and further elucidation of the correlation between BTK and macrophages in atherogenesis is expected.

Here, we elucidated that BTK knockdown restrained ox-LDL-induced NK-*κ*B signaling in macrophages and repressed the M1 polarization but promoted the M2 polarization of macrophages. Moreover, we discovered that oxidative stress, mitochondrial injury, and ER stress in ox-LDL-stimulated macrophages were also regulated after inhibiting BTK. A well-established diet-induced mouse model that mimics human atherogenesis is recognized as a critical methodology to understand the function and mechanism of potential targets in human AS [25]. Therefore, we elucidated that the severity of AS in ApoE^−/−^ mice induced by a high-fat diet was alleviated by the sh-BTK adenovirus injection, suggesting that BTK may represent a potential therapeutic target to combat AS.

## 2. Materials and Methods

### 2.1. Clinical Samples

We collected the clinical specimens from 8 females and 7 males, with an average age of 54.6 ± 9.7 years, who accepted coronary bypass surgery from 2018 to 2020. The coronary arteries with AS plaques and the internal mammary arteries were gathered. The study was approved by the Ethical Committee of Sun Yat-sen University, Sun Yat-sen Memorial Hospital (SYSEC-KY-KS2020-090).

### 2.2. Cell Line

RAW264.7 macrophages (CL-0190, Procell), which were maintained at 37°C in a humidified 5% CO_2_ incubator, grew in high-glucose DMEM, with 10% fetal bovine serum and penicillin (100 U/ml)-streptomycin (0.1 mg/ml). Twenty-four hours before further experiments, the cells would be seeded in culture dishes or 6-well plates [26, 27].

### 2.3. Small Interfering RNA (siRNA) and Treatment

The siRNA was designed and synthesized by RiboBio, China. Macrophages were seeded at a concentration of 5 × 10^5^ cells per well in 6-well plates. After 24 hours, macrophages were added with BTK-siRNA with the RNAiMAX reagent (13778075, Thermo Fisher Scientific). The transfected macrophages were collected after 48 hours. The ox-LDL (Cat. No. YB-002, Yiyuan Biotech) was used at a concentration of 50 *μ*g/ml as previously described [28, 29].

### 2.4. PPI Network Establishment and Hub Gene Identification

The raw CEL data of the GSE70126 dataset was downloaded from the GEO database of NCBI. And the bioinformatic analysis was performed essentially as described by Qiu et al. [30]. Briefly, STRING and Cytoscape software programs were used to construct the protein-protein interaction (PPI) network and screen differentially expressed genes. MCODE and cytoHubba applications of Cytoscape, as well as the Metascape online tool, were applied (https://metascape.org) [31, 32].

### 2.5. qRT-PCR

Total RNA was isolated from RAW264.7 macrophages using RNAiso Plus (TaKaRa) [33, 34]. RNA was converted into cDNA using PrimeScript RT Mix (TaKaRa). Afterward, qRT-PCR experiments were conducted in a LightCycler PCR system (Roche) with UNICONTM SYBR Green (11198ES08, Yeasen). The sequences of all the primers used are shown in Table 1.

### 2.6. Western Blot and ELISA

The experiments of Western blot and ELISA were performed essentially as described in Li et al. [35]. Shortly, RAW264.7 macrophages were lysed with the RIPA buffer to obtain total protein, additionally with a nuclear and cytoplasmic extraction kit (CWbiotech) according to the manufacturer's protocol. Proteins were quantified with a bicinchoninic acid (BCA) assay kit (Thermo Fisher Scientific) and analyzed by SDS-PAGE with 10% polyacrylamide gel. The primary antibodies of NRF2, histone H3, p-PERK, p-IRE1, BTK, p-p65, p-IRF3, DRP1, FIS1, and *β*-actin (Cell Signaling Technology) were used. An enhanced chemiluminescence (ECL) reagent detection kit (Thermo Fisher Scientific) was used, and an imaging machine (2200 Pro, Kodak) was employed to detect the protein bands. For ELISA, the supernatants of RAW264.7 macrophages were collected to detect the concentration of TNF-*α* and IL-6 (Neobioscience, China) [36, 37].

### 2.7. Flow Cytometry and ROS Detection

M1 and M2 macrophage polarization was detected by their makers, iNOS and COX-2, respectively. In brief, the macrophages with different treatments were incubated with the iNOS antibody or COX-2 antibody for 20 min. Then, the cells were trypsinized, washed with PBS, and resuspended in PBS. A DHE-DA staining kit (KGAF019, KeyGen Biotech) was used for analyzing the production of ROS as described before [38, 39]. Flow cytometry (BD FACSVerse) was applied to detect M1 polarization, M2 polarization, and ROS production [40, 41].

### 2.8. MitoTracker Staining

The macrophages were stained with MitoTracker Red (Beyotime) for the analysis of change in mitochondrial injury. Macrophages were cultured in 96-well plates and incubated for 30 minutes with MitoTracker Red. Then, the macrophages were counterstained with DAPI for 10 min and visualized by fluorescence microscopy (LSM 710, Carl Zeiss) [42, 43].

### 2.9. Immunofluorescent Staining and Confocal Microscopy

The experiments of immunofluorescent staining and confocal microscopy were performed essentially as described in Fang et al. [44]. Shortly, macrophages were seeded in confocal dishes fixed with 4% paraformaldehyde after different treatments. After being blocked by goat serum, RAW264.7 macrophages were incubated with different primary antibodies (p65, ATF6, TNF-*α*, and IL-6; Cell Signaling Technology) overnight at 4°C. A secondary antibody (goat anti-rabbit, Abcam) and a DAPI-containing Anti-Fade Fluorescence Mounting Medium (HelixGen Co., Ltd.) were used for visualization with confocal microscopy (Carl Zeiss) [45].

### 2.10. Immunohistochemistry Assay

The sections of the mouse hearts with aortic roots were heated for 2 h, then dewaxed with xylene and dehydrated with ethanol. An H&E staining kit (AR1180, Boster Biological Technology Co., Ltd.) was used, as described by Qiu et al. [30]. For the observation of the sections of mouse aortic roots, a Leica biomicroscope was employed [46].

### 2.11. Mouse Atherosclerosis Model Establishment

The mouse AS model was established as described in our previous study [28]. Briefly, ApoE^−/−^ mice (males, 8 weeks old, and 20 g body weight) with C57BL/6J background were purchased from GemPharmatech Co., Ltd. All mice were reared in a specific pathogen-free environment with a 12 h light/12 h dark cycle at the temperature of 22 ± 2°C and the relative humidity of 40-55%. ApoE^−/−^ mice were fed a diet containing 21% wt/wt saturated fat and 0.2% wt/wt cholesterol (Western diet) for 12 weeks for the establishment of the AS model. Recombinant adenovirus for the mouse *Btk* interference (sh-BTK adenovirus), alone with sh-NC adenovirus as the control, was synthesized by GeneChem Co., Ltd. The Western diet-fed mice have been applied with a tail vein injection of adenovirus. All the animal procedures were approved by the Laboratory Animal Welfare and Ethics Committee of Sun Yat-sen University.

### 2.12. Atherosclerotic Lesion Analysis

Mice were anesthetized, and the mouse hearts with aortic roots as well as whole aortas were harvested. For inner face plaque analyses, the whole aortas without adipose tissue, fixed in 4% paraformaldehyde overnight, were stained with Oil Red O solution for 15 min and washed with 70% ethanol. For aortic root cross-section analysis, the mouse hearts with aortic roots were embedded and sliced into several sections, followed by staining with Oil Red O solution as described in our previous study [28]. Images were viewed by a biomicroscope (DM200, Leica) [47, 48]. The percentage of Oil Red O-positive areas, presumed to be atherosclerotic lesions, was quantified by ImageJ software.

### 2.13. Statistical Analysis

Three independent experiments were carried out. Data were analyzed to verify data normality using Kolmogorov-Smirnov tests and are expressed as mean ± standard deviation (SD). A two-sided Student's *t*-test was performed between two groups. And one-way analysis of variance (ANOVA) and Fisher's least significant difference (LSD) test were utilized among three or more than three groups. The threshold of statistical significance was a *P* value of <0.05.

## 3. Results

### 3.1. Oxidative Stress, Mitochondrial Injury, and ER Stress Were Triggered by ox-LDL in Macrophages

**Figure 1:**
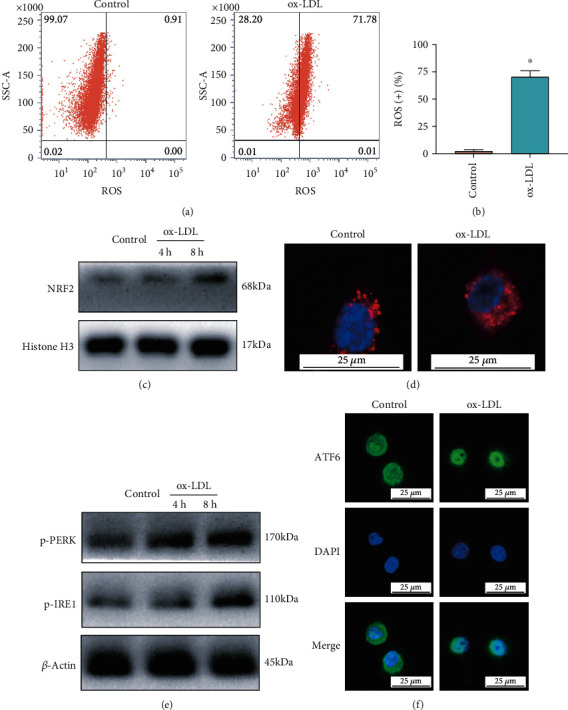
ox-LDL triggers oxidative stress, mitochondrial injury, and ER stress in macrophages. (a, b) The number of ROS-positive macrophages increased after ox-LDL treatment for 24 h. (c) The expression levels of nuclear NRF2 by Western blot in macrophages after ox-LDL stimulation for 4 h and 8 h. (d) Mitochondria-specific fluorescence probes (MitoTracker Red) in macrophages captured by confocal microscopy. (e) The phosphorylation levels of PERK and IRE1 in macrophages after ox-LDL stimulation for 4 h and 8 h. (f) ATF6 protein (green) translocated into the nucleus after ox-LDL treatment in macrophages. The threshold of statistical significance was set at ^∗^*P* < 0.05.

To investigate the underlying mechanism by which ox-LDL accelerates AS, we stimulated macrophages with ox-LDL for different periods. We then found that the number of ROS-positive macrophages significantly increased after ox-LDL treatment for 24 h (Figures 1(a) and 1(b)). Furthermore, the nucleus protein level of NRF2 in macrophages increased after 4 h and 8 h of stimulation for ox-LDL (Figure 1(c)). Concurrently, the mitochondria-specific fluorescence probe (MitoTracker Red) was used, and the result showed that ox-LDL induced the structural disorder of mitochondria in macrophages, suggesting an obvious mitochondrial injury (Figure 1(d)). As for ER stress, we found that ox-LDL could induce the phosphorylation of PERK and IRE1 (Figure 1(e)). Moreover, our fluorescent staining results showed that ATF6 protein (green) translocated into the nucleus after ox-LDL treatment in macrophages (Figure 1(f)). The activation of these 3 unfolded protein response (UPR) activator proteins indicated that ox-LDL triggered ER stress in macrophages.

### 3.2. BTK Was the Hub Gene Related to Oxidative Stress, ER Stress, and Inflammation in Macrophage-Induced AS

**Figure 2:**
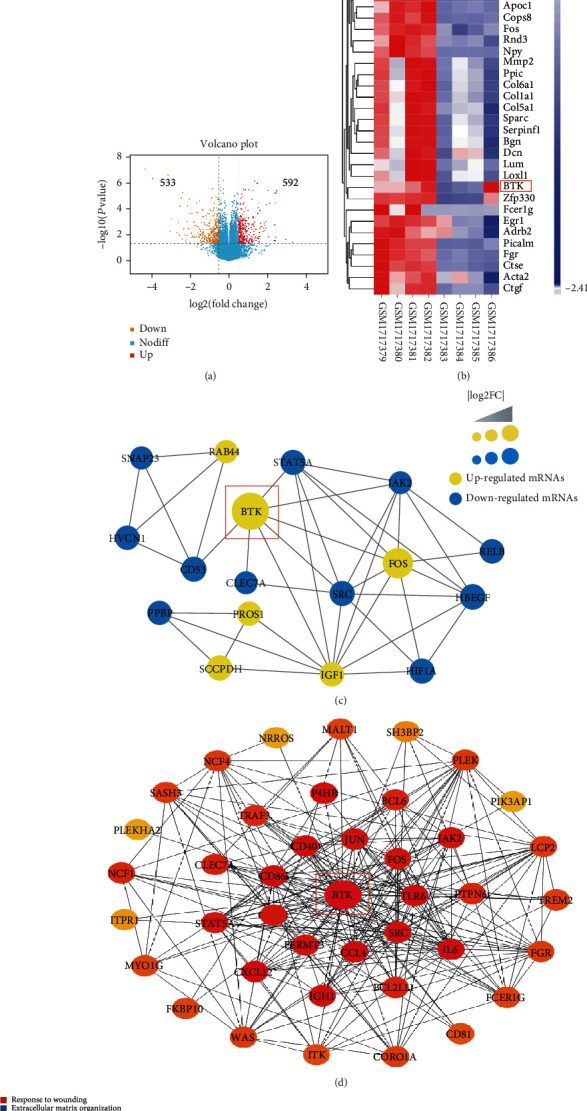
BTK was determined as the hub gene related to oxidative stress, ER stress, and inflammation in macrophage-induced AS. (a) The volcano plot indicated 592 upregulated DEGs and 533 downregulated DEGs. (b) The heat map and cluster analysis. (c) STRING and Cytoscape software programs were used to construct the PPI network, and we obtained 19 hub genes through the MCODE application, of which BTK had a high score. (d) By using the cytoHubba application, the hub gene network following core gene scores was built. (e) The functional clustering network from the Metascape database indicated that BTK was closely related to oxidative stress, ER stress, and inflammation.

To assess the potential hub genes in atherosclerosis induced by macrophages, the differentially expressed genes (DEGs) from the GSE70126 dataset were obtained. We found 1125 differentially expressed genes in foam cell macrophages, as compared with nonfoamy macrophages: 592 were upregulated, and 533 were downregulated. The volcano plot (Figure 2(a)) and the heat map (Figure 2(b)) were shown, respectively. STRING and Cytoscape software programs were used to construct the PPI network, and we obtained 19 hub genes through the MCODE application, of which BTK had a high score (Figure 2(c)). Besides, by using the cytoHubba application, we built the hub gene network following core gene scores (Figure 2(d)). Furthermore, the functional clustering network from the Metascape database indicated that BTK was closely related to oxidative stress, ER stress, and inflammation (Figure 2(e)).

### 3.3. BTK Knockdown Suppressed the Inflammatory Responses of ox-LDL-Induced Macrophages

**Figure 3:**
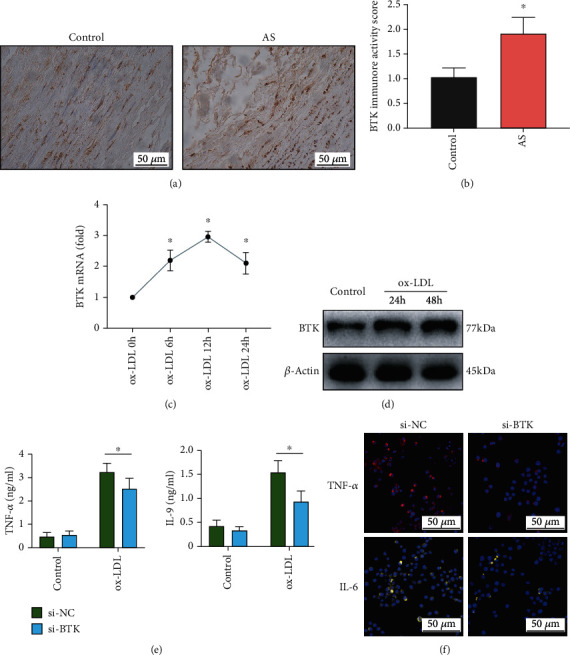
BTK knockdown suppressed the inflammatory responses of ox-LDL-induced macrophages. (a) IHC staining and (b) immunoreactivity score of BTK in specimens from the control group and AS group. (c, d) The mRNA and protein expression levels of BTK were upregulated in ox-LDL-induced macrophages. (e) The siRNA-mediated inhibition of BTK was performed in macrophages, and the ELISA results indicated that TNF-*α* and IL-6 were reduced by BTK knockdown. (f) The fluorescent intensities of TNF-*α* and IL-6 were restrained by the knockdown of BTK in ox-LDL-induced macrophages. The threshold of statistical significance was set at ^∗^*P* < 0.05.

To explore the relationship between the BTK gene and AS, immunohistochemical staining was performed. A high cytoplasm protein level of BTK was found in the lesion area of the aortas from the AS patients (Figures 3(a) and 3(b)). Besides, we stimulated macrophages with ox-LDL for different periods (6, 12, and 24 h for BTK qRT-PCR; 24 and 48 h for BTK Western blot; 48 h for proinflammatory factor ELISA; and 24 h for fluorescent staining). The expression level of BTK was upregulated in ox-LDL-induced macrophages, including mRNA and protein expression (Figures 3(c) and 3(d)). Moreover, siRNA-mediated inhibition of BTK was performed in macrophages, and the ELISA results indicated that BTK knockdown could attenuate the cytokine levels of TNF-*α* and IL-6 (Figure 3(e)). Consistent with the ELISA results, the fluorescent intensities of TNF-*α* and IL-6 in ox-LDL-induced macrophages were also restrained by the knockdown of BTK (Figure 3(f)).

### 3.4. BTK Knockdown Suppressed the ox-LDL-Induced NK-*κ*B Signaling in Macrophages, Inhibited M1 Macrophage Polarization, and Facilitated M2 Polarization

**Figure 4:**
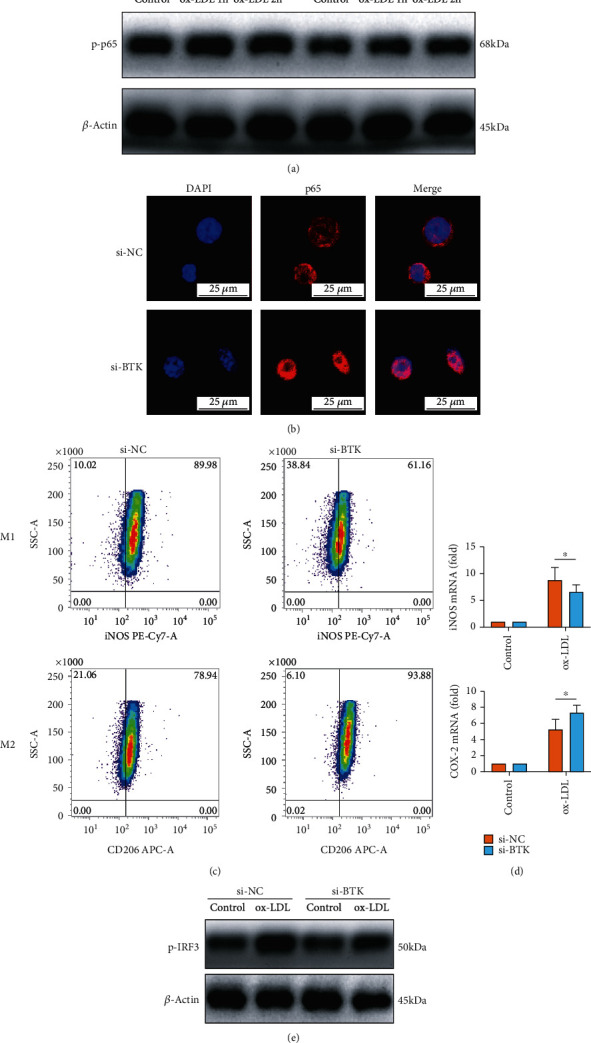
BTK knockdown suppressed the ox-LDL-induced NK-*κ*B activation and inhibited M1 polarization. (a) The phosphorylation level of p65 protein was significantly reduced by the knockdown of BTK. (b) BTK knockdown inhibited the nucleus translocation of p65 in macrophages. (c) BTK knockdown reduced the number of M1 macrophages and significantly increased the M2 anti-inflammatory macrophages. (d) iNOS was downregulated, while COX-2 was upregulated on the mRNA level caused by BTK knockdown. (e) The knockdown of BTK significantly reduced the protein expression level of phosphorylated IRF3. The threshold of statistical significance was set at ^∗^*P* < 0.05.

As shown in Figure 4(a), the phosphorylation level of p65 protein, an important NK-*κ*B signaling mediator, was significantly reduced by the knockdown of BTK. Moreover, we performed the fluorescent staining of p65 protein, and the results indicated that BTK knockdown inhibited the nucleus translocation of p65 in macrophages (Figure 4(b)). Macrophage polarization, which is closely related to the NF-*κ*B pathway, was also determined by flow cytometry here. As shown in Figure 4(c), BTK knockdown decreased the number of M1 macrophages, which were considered a proinflammatory type. In contrast, M2 anti-inflammatory macrophages significantly increased caused by the knockdown of BTK. Besides, the expression of iNOS was downregulated, while the expression of COX-2 was upregulated on the mRNA level caused by BTK knockdown (Figure 4(d)). Consistently, the knockdown of BTK significantly reduced the protein expression level of phosphorylated IRF3, which is an important transcription factor in the process of M1 polarization (Figure 4(e)).

### 3.5. BTK Knockdown Alleviated Oxidative Stress, Mitochondrial Injury, and ER Stress in ox-LDL-Induced Macrophages

**Figure 5:**
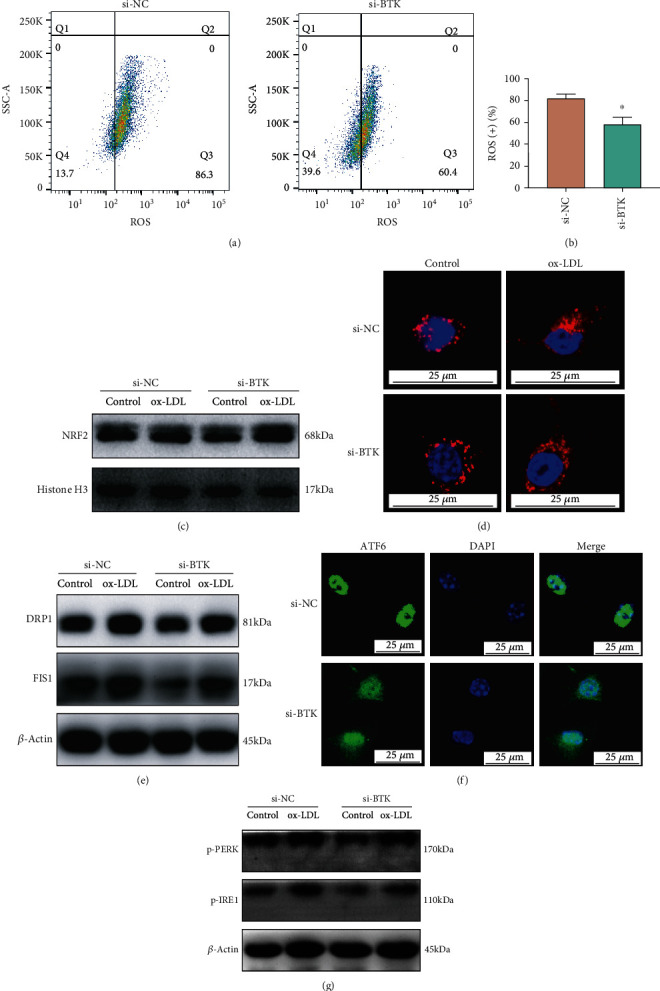
BTK knockdown alleviated oxidative stress, mitochondrial injury, and ER stress in ox-LDL-induced macrophages. (a, b) The flow cytometry results showed that BTK knockdown significantly reduced the production levels of ROS. (c) The upregulation of the NRF2 nuclear protein level in ox-LDL-stimulated macrophages when BTK was knocked down. (d) BTK knockdown attenuated the mitochondrial injury in macrophages. (e) The knockdown of BTK suppressed the protein expression of DRP1 and FIS1. (f) The confocal microscope results revealed that ox-LDL-induced nuclear translocation of ATF6 was attenuated by BTK knockdown. (g) The phosphorylation levels of PERK and IRE1 significantly decreased in the si-BTK group. The threshold of statistical significance was set at ^∗^*P* < 0.05.

In view of the ox-LDL-triggered oxidative stress that played an important role in AS, we next determined the effect of BTK knockdown on oxidative stress and mitochondrial injury. Flow cytometry results revealed that BTK knockdown significantly reduced the production levels of ROS (Figures 5(a) and 5(b)). The upregulation of the NRF2 nuclear protein level in ox-LDL-stimulated macrophages when BTK was knocked down is shown in Figure 5(c). Moreover, BTK knockdown attenuated the mitochondrial injury in macrophages, as shown in our fluorescent staining results (Figure 5(d)). Besides, the knockdown of BTK clearly suppressed the protein expression of DRP1 and FIS1, the two important mitochondrial fission factors (Figure 5(e)). To further determine the correlation between BTK knockdown and ER stress, we administered ox-LDL to macrophages for different periods. A fluorescent staining assay was carried out to clarify the activation of ATF6 protein, an ER stress-related transcription factor. The confocal microscope results showed that ox-LDL-stimulated nuclear translocation of ATF6 was attenuated by BTK knockdown (Figure 5(f)). Besides, the results demonstrated that the phosphorylation levels of PERK and IRE1 significantly decreased in the si-BTK group (Figure 5(g)).

### 3.6. sh-BTK Adenovirus Injection Reduced the Progression of AS Lesions in ApoE^−/−^ Mice

**Figure 6:**
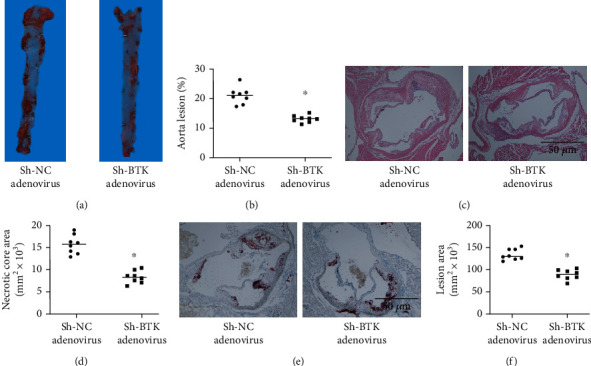
sh-BTK adenovirus injection reduced the development of AS lesions in ApoE^−/−^ mice. (a, b) The Oil Red O area within the aortic tree was significantly attenuated in the sh-BTK group. The lesion coverage of the entire aorta (%) was assessed by ImageJ software. (c, d) The H&E staining results showed that the necrotic core area in ApoE^−/−^ mice decreased in the sh-BTK group. The necrotic core area was assessed by ImageJ software. (e, f) The lesion area significantly decreased in the sh-BTK group. The lesion area was assessed by ImageJ software. The threshold of statistical significance was set at ^∗^*P* < 0.05.

The therapeutic roles of BTK in atherogenesis were determined in ApoE^−/−^ mice, which were fed with a Western diet for 12 weeks. Then, the tail vein injection of adenovirus-mediated sh-BTK and sh-NC was performed. We observed that the Oil Red O area within the aortic tree was significantly attenuated in the sh-BTK group, which indicated that sh-BTK adenovirus injection effectively attenuated the progression of AS (Figures 6(a) and 6(b)). Consistently, the H&E staining results showed that the necrotic core area in ApoE^−/−^ mice decreased in the sh-BTK group (Figures 6(c) and 6(d)). Besides, the foam cells in the cross-sections of ApoE^−/−^ mouse aortas were stained with Oil Red O, and the results showed that the lesion area significantly decreased in the sh-BTK group (Figures 6(e) and 6(f)). Hence, our results indicated that the knockdown of BTK reduced the development of AS in ApoE^−/−^ mice.

## 4. Discussion

AS is a progressive chronic inflammatory condition shared by several cardiovascular diseases, bringing immense burdens on health and economy worldwide [49, 50]. Using genetically engineered mice, our results suggested that the knockdown of BTK inhibited the development of AS in ApoE^−/−^ mice, and one of the mechanisms is through releasing ox-LDL-induced inflammatory response, oxidative stress, and ER stress in macrophages and promoting macrophage polarization to the M2 type. It is well recognized that the biochemical cascade of atherogenesis is inseparable from the foam cell formation and inflammatory responses of macrophages [51]. ox-LDL, an atherogenic factor, contributes to AS progression by promoting the inflammatory response in macrophages [52]. Consistently, we found that the NF-*κ*B signaling pathway and IRF3 were activated by ox-LDL in macrophages. NF-*κ*B was considered a first responder to regulate the central cellular response to inflammation [19]. And activation of the interferon regulatory factor 3 (IRF3), a downstream factor that induced the type I interferon production and initiated inflammation, suggested the increase of the ox-LDL-induced M1 phenotype macrophages [53]. Inflammation and polarization of macrophages play an important role in AS. In our previous report, we also found that atherosclerotic lesions can be alleviated when inflammatory responses of ox-LDL-induced macrophages were released [28].

Nevertheless, the exact mechanisms of how ox-LDL stimulated macrophages have not been clarified. Single-cell RNA-seq of aortic macrophages in murine AS identified that inflammatory macrophages (enriched in M1 proinflammatory genes) accounted for a large part of the overall macrophage subpopulations [54]. In our current study, RNA-seq analysis has been applied in foam cell macrophages. After analysis of DEGs and the PPIN, we performed a detailed comparison of gene expression. BTK was considered a hub gene related to oxidative stress, ER stress, and inflammation in macrophage-induced AS. Consistently, we showed that the protein level of BTK in the AS group increased significantly than that in the control group, and the knockdown of BTK significantly suppressed the proinflammatory factor secretion and the phosphorylation level of p65 protein, indicating that BTK positively regulated the inflammatory responses of ox-LDL-stimulated macrophages. Thus, these results implicated BTK as a candidate causal gene for AS and pointed to future directions to treatment for AS, using pharmacological or genetic inhibition of BTK.

Former investigators indicated that BTK positively regulated TLR4-mediated activation of the NF-*κ*B signaling pathway in the monocytic cell line [15, 55]. These data are consistent with our results that BTK knockdown decreased NF-*κ*B signaling pathway activation in ox-LDL-induced macrophages. Another study reported that BTK also made sense on lipopolysaccharide-induced sepsis and negatively regulated the lipopolysaccharide-induced canonical NF-*κ*B signaling pathway in mast cells [56]. This discrepancy may be caused by different cell types and stimuli. The role of BTK might be cell type-specific. The activation of the NF-*κ*B signaling pathway and the secretion of proinflammatory factors have a strong association with the macrophage polarization to the M1 type [57]. Since BTK positively regulates the NF-*κ*B signaling pathway in our current study, BTK knockdown inhibited M1 macrophage polarization and facilitated M2 polarization, which was validated by the flow cytometry analysis. Phosphorylation levels of IRF3 were significantly decreased accompanied by fewer M1 macrophages.

Previous studies have shown that ER stress and oxidative stress were extremely significant in macrophage polarization and proinflammatory factor secretion [58–60]. ER stress, oxidative stress, and inflammatory response together make up defense networks of the cells that react to outside stimuli [8, 61]. Risk factors related to AS, such as hyperlipidemia, hypertension, and obesity, activated the ER stress-related proteins, such as IRE1, PERK, and ATF6, and aggravated the inflammatory response in macrophages [62, 63]. UPR activation and ER stress had been identified in atherosclerotic lesions. ER stress inhibitors could relieve inflammation and decreased IL-6 secretion in macrophages via inhibition of the ER stress-related PERK-AFT4 pathway [7]. Additionally, AS development was closely related to oxidative stress and mitochondrial injury. Suppression of mitochondrial oxidative stress in macrophages attenuated AS in mice. The imbalanced mitochondrial homeostasis and excessive ROS production in macrophages would activate the NF-*κ*B signaling pathway and promote the release of proinflammatory factors such as TNF-*α* and IL-6 [10, 64]. The interaction between ROS and proinflammatory factors would also enhance the damage and aggravate the inflammation, implicating the pathogenesis of the atherosclerotic lesion [11]. Researchers revealed that MAMs, associated with the mitochondria with ER, made sense on several metabolic diseases, including diabetes, stroke, and coronary artery diseases [65, 66]. And this association between ER and mitochondria was considered a potential target for many cardiovascular diseases. We show in the current study that the knockdown of BTK could attenuate the ER stress and mitochondrial oxidative stress. Our findings advanced our understanding of the association between MMAs and metabolic disorders in AS.

Finally, we had reported that the injection of ZBTB20 knockdown adenovirus in the tail vein of ApoE^−/−^ mice fed with a Western diet could alleviate AS, indicating that the tail vein adenovirus injection is a potential method for treatment of cardiovascular diseases [28]. In our current study, ApoE^−/−^ mice injected with BTK knockdown adenovirus presented a smaller AS lesion area in the aorta. BTK knockdown attenuates the progression of AS and foam cell formation in mice, suggesting that BTK positively regulates the development of AS in ApoE^−/−^ mice fed with the Western diet. Despite our rigorous experimental design, there are some limitations in our study. Although adenovirus vectors have recently emerged as attractive vectors for cardiovascular gene therapy due to their high transduction efficiency, direct distribution of the adenovirus in mice could not be identified. Moreover, adenovirus tail vein injection cannot definitively prove the effector cell types. Therefore, the cell-specific BTK knockdown ApoE^−/−^ mice need to be addressed in future research. The present study showed that the knockdown of the BTK led to the attenuation of the oxidative stress, mitochondrial stress, ER stress, and inflammation in macrophages induced by ox-LDL, which were consistent with the decreased ROS production levels, alleviated mitochondrial injury, inhibited ATF6 nuclear translocation, decreased M1 macrophages, and reduced proinflammatory cytokine secretion. Additionally, the progression of AS of the Western diet-fed ApoE^−/−^ mice was attenuated by intravenous injection of BTK knockdown adenovirus, suggesting that BTK inhibitors may make a difference in AS treatment as a powerful therapeutic target.

All in all, we clarified that BTK knockdown reduced the AS lesion area and attenuated the progression of AS in mice, and BTK positively regulated inflammatory responses of ox-LDL-induced macrophages in AS by regulating oxidative stress, mitochondrial injury, and ER stress.

## Figures and Tables

**Table 1:** Primers.

BTK	Forward	GAGGAGAGGTGAGGAGTCTAGT
	Reverse	AGCTCTTCAGTTGGGGAGAAAA
iNOS	Forward	GGAGTGACGGCAAACATGACT
	Reverse	TCGATGCACAACTGGGTGAAC
COX-2	Forward	TGCACTATGGTTACAAAAGCTGG
	Reverse	TCAGGAAGCTCCTTATTTCCCTT
GAPDH	Forward	TGTGTCCGTCGTGGATCTGA
	Reverse	TTGCTGTTGAAGTCGCAGGAG

## Data Availability

All the datasets were available from the corresponding authors.

## References

[1] Kim H. W., Shi H., Winkler M. A., Lee R., Weintraub N. L. (2020). Perivascular adipose tissue and vascular perturbation/atherosclerosis. *Arteriosclerosis, Thrombosis, and Vascular Biology*.

[2] Shah P. K. (2019). Inflammation, infection and atherosclerosis. *Trends in Cardiovascular Medicine*.

[3] Khallou-Laschet J., Varthaman A., Fornasa G. (2010). Macrophage plasticity in experimental atherosclerosis. *PLoS One*.

[4] Galle-Treger L., Moreau M., Ballaire R. (2020). Targeted invalidation of SR-B1 in macrophages reduces macrophage apoptosis and accelerates atherosclerosis. *Cardiovascular Research*.

[5] Mueller P. A., Zhu L., Tavori H. (2018). Deletion of macrophage low-density lipoprotein receptor-related protein 1 (LRP1) accelerates atherosclerosis regression and increases C-C chemokine receptor type 7 (CCR7) expression in plaque macrophages. *Circulation*.

[6] Wang R., Zhang Y., Xu L. (2016). Protein Inhibitor of Activated STAT3 Suppresses Oxidized LDL-induced Cell Responses during Atherosclerosis in Apolipoprotein E-deficient Mice. *Scientific Reports*.

[7] Chen S., Chen Y., Chen Y., Yao Z. (2019). InP/ZnS quantum dots cause inflammatory response in macrophages through endoplasmic reticulum stress and oxidative stress. *International Journal of Nanomedicine*.

[8] Dandekar A., Mendez R., Zhang K. (2015). Cross talk between ER stress, oxidative stress, and inflammation in health and disease. *Methods in Molecular Biology*.

[9] Liao Y., Hussain T., Liu C. (2019). Endoplasmic reticulum stress induces macrophages to produce IL-1*β* during *Mycobacterium bovis* infection via a positive feedback loop between mitochondrial damage and inflammasome activation. *Frontiers in Immunology*.

[10] Madamanchi N. R., Runge M. S. (2007). Mitochondrial dysfunction in atherosclerosis. *Circulation Research*.

[11] Missiroli S., Patergnani S., Caroccia N. (2018). Mitochondria-associated membranes (MAMs) and inflammation. *Cell Death & Disease*.

[12] Momtazi-Borojeni A. A., Abdollahi E., Nikfar B., Chaichian S., Ekhlasi-Hundrieser M. (2019). Curcumin as a potential modulator of M1 and M2 macrophages: new insights in atherosclerosis therapy. *Heart Failure Reviews*.

[13] Liu Y., Wang X., Pang J. (2019). Attenuation of Atherosclerosis by Protocatechuic Acid via Inhibition of M1 and Promotion of M2 Macrophage Polarization. *Journal of Agricultural and Food Chemistry*.

[14] Afaghani J., Taylor J. (2021). A moving target: inactivating BTK mutations as drivers of follicular lymphoma. *Clinical Cancer Research*.

[15] Yue C., Niu M., Shan Q. Q. (2017). High expression of Bruton’s tyrosine kinase (BTK) is required for EGFR-induced NF-*κ*B activation and predicts poor prognosis in human glioma. *Journal of Experimental & Clinical Cancer Research*.

[16] Bhargava P., Kim S., Reyes A. A. (2021). Imaging meningeal inflammation in CNS autoimmunity identifies a therapeutic role for BTK inhibition. *Brain*.

[17] Pontoriero M., Fiume G., Vecchio E. (2019). Activation of NF-*κ*B in B cell receptor signaling through Bruton’s tyrosine kinase-dependent phosphorylation of I*κ*B-*α*. *Journal of Molecular Medicine (Berlin, Germany)*.

[18] Choi J., Phelan J. D., Wright G. W. (2020). Regulation of B cell receptor-dependent NF-*κ*B signaling by the tumor suppressor KLHL14. *Proceedings of the National Academy of Sciences*.

[19] Pateras I., Giaginis C., Tsigris C., Patsouris E., Theocharis S. (2014). NF-*κ*B signaling at the crossroads of inflammation and atherogenesis: searching for new therapeutic links. *Expert Opinion on Therapeutic Targets*.

[20] Papin A., Tessoulin B., Bellanger C. (2019). CSF1R and BTK inhibitions as novel strategies to disrupt the dialog between mantle cell lymphoma and macrophages. *Leukemia*.

[21] Colado A., Genoula M., Cougoule C. (2018). Effect of the BTK inhibitor ibrutinib on macrophage- and *γδ* T cell-mediated response against *Mycobacterium tuberculosis*. *Blood Cancer Journal*.

[22] Feng M., Chen J. Y., Weissman-Tsukamoto R. (2015). Macrophages eat cancer cells using their own calreticulin as a guide: roles of TLR and Btk. *Proceedings of the National Academy of Sciences of the United States of America*.

[23] Herbst S., Shah A., Mazon Moya M. (2015). Phagocytosis-dependent activation of a TLR9-BTK-calcineurin-NFAT pathway co-ordinates innate immunity to Aspergillus fumigatus. *EMBO Molecular Medicine*.

[24] Busygina K., Jamasbi J., Seiler T. (2018). Oral Bruton tyrosine kinase inhibitors selectively block atherosclerotic plaque-triggered thrombus formation in humans. *Blood*.

[25] von Scheidt M., Zhao Y., Kurt Z. (2017). Applications and limitations of mouse models for understanding human atherosclerosis. *Cell Metabolism*.

[26] Bai J., Khajavi M., Sui L. (2021). Angiogenic responses in a 3D micro-engineered environment of primary endothelial cells and pericytes. *Angiogenesis*.

[27] Abbas N., Perbellini F., Thum T. (2020). Non-coding RNAs: emerging players in cardiomyocyte proliferation and cardiac regeneration. *Basic Research in Cardiology*.

[28] Tao J., Qiu J., Lu L. (2021). ZBTB20 Positively Regulates Oxidative Stress, Mitochondrial Fission, and Inflammatory Responses of ox-LDL-Induced Macrophages in Atherosclerosis. *Oxidative Medicine and Cellular Longevity*.

[29] Adapala R. K., Kanugula A. K., Paruchuri S., Chilian W. M., Thodeti C. K. (2020). TRPV4 deletion protects heart from myocardial infarction-induced adverse remodeling via modulation of cardiac fibroblast differentiation. *Basic Research in Cardiology*.

[30] Qiu J., Peng P., Xin M. (2020). ZBTB20-mediated titanium particle-induced peri-implant osteolysis by promoting macrophage inflammatory responses. *Biomaterials Science*.

[31] Bakhta O., Pascaud A., Dieu X. (2020). Tryptophane–kynurenine pathway in the remote ischemic conditioning mechanism. *Basic Research in Cardiology*.

[32] Li L., Bi Z., Hu Y. (2021). Silver nanoparticles and silver ions cause inflammatory response through induction of cell necrosis and the release of mitochondria in vivo and in vitro. *Cell Biology and Toxicology*.

[33] Bridges E., Sheldon H., Kleibeuker E. (2020). RHOQ is induced by DLL4 and regulates angiogenesis by determining the intracellular route of the Notch intracellular domain. *Angiogenesis*.

[34] Wang J., Zhou H. (2020). Mitochondrial quality control mechanisms as molecular targets in cardiac ischemia–reperfusion injury. *Acta Pharmaceutica Sinica B*.

[35] Li S., Qiu J., Qin L. (2019). NOD2 negatively regulated titanium particle-induced osteolysis in mice. *Biomaterials Science*.

[36] Buglak D. B., Kushner E. J., Marvin A. P., Davis K. L., Bautch V. L. (2020). Excess centrosomes disrupt vascular lumenization and endothelial cell adherens junctions. *Angiogenesis*.

[37] Bekhite M. M., Delgado A. G., Menz F. (2020). Longitudinal metabolic profiling of cardiomyocytes derived from human-induced pluripotent stem cells. *Basic Research in Cardiology*.

[38] Tan Y., Mui D., Toan S., Zhu P., Li R., Zhou H. (2020). SERCA overexpression improves mitochondrial quality control and attenuates cardiac microvascular ischemia-reperfusion injury. *Molecular Therapy-Nucleic Acids*.

[39] Wang J., Zhu P., Li R., Ren J., Zhou H. (2020). Fundc1-dependent mitophagy is obligatory to ischemic preconditioning-conferred renoprotection in ischemic AKI via suppression of Drp1-mediated mitochondrial fission. *Redox Biology*.

[40] Cao F., Maguire M. L., McAndrew D. J. (2020). Overexpression of mitochondrial creatine kinase preserves cardiac energetics without ameliorating murine chronic heart failure. *Basic Research in Cardiology*.

[41] Wang J., Toan S., Li R., Zhou H. (2020). Melatonin fine-tunes intracellular calcium signals and eliminates myocardial damage through the IP3R/MCU pathways in cardiorenal syndrome type 3. *Biochemical Pharmacology*.

[42] Lobo-Gonzalez M., Galán-Arriola C., Rossello X. (2020). Metoprolol blunts the time-dependent progression of infarct size. *Basic Research in Cardiology*.

[43] Dudley A. C. (2020). Introduction to special issue: vascular co-option in cancer. *Angiogenesis*.

[44] Fang G., Fu Y., Li S. (2020). The USP14-NLRC5 pathway inhibits titanium particle-induced osteolysis in mice by suppressing NF-*κ*B and PI3K/AKT activities. *The Journal of Biological Chemistry*.

[45] Fournier P., Viallard C., Dejda A., Sapieha P., Larrivée B., Royal I. (2020). The protein tyrosine phosphatase PTPRJ/DEP-1 contributes to the regulation of the Notch-signaling pathway and sprouting angiogenesis. *Angiogenesis*.

[46] Lindner M., Mehel H., David A. (2020). Fibroblast growth factor 23 decreases PDE4 expression in heart increasing the risk of cardiac arrhythmia; Klotho opposes these effects. *Basic Research in Cardiology*.

[47] Lahiri S. K., Quick A. P., Samson-Couterie B. (2020). Nuclear localization of a novel calpain-2 mediated junctophilin-2 C-terminal cleavage peptide promotes cardiomyocyte remodeling. *Basic Research in Cardiology*.

[48] Ludwig N., Yerneni S. S., Azambuja J. H. (2020). Tumor-derived exosomes promote angiogenesis via adenosine A_2B_ receptor signaling. *Angiogenesis*.

[49] Authors/Task Force members, Erbel R., Aboyans V. (2014). 2014 ESC Guidelines on the diagnosis and treatment of aortic diseases. *European Heart Journal*.

[50] Benjamin E. J., Blaha M. J., Chiuve S. E. (2017). Correction to: Heart Disease and Stroke Statistics—2017 Update: A Report From the American Heart Association. *Circulation*.

[51] Wang D., Yang Y., Lei Y. (2019). Targeting foam cell formation in atherosclerosis: therapeutic potential of natural products. *Pharmacological Reviews*.

[52] Pirillo A., Norata G. D., Catapano A. L. (2013). LOX-1, OxLDL, and Atherosclerosis. *Mediators of Inflammation*.

[53] Chen X., Xu Y., Tu W. (2021). Ubiquitin E3 ligase MID1 inhibits the innate immune response by ubiquitinating IRF3. *Immunology*.

[54] Cochain C., Vafadarnejad E., Arampatzi P. (2018). Single-cell RNA-seq reveals the transcriptional landscape and heterogeneity of aortic macrophages in murine atherosclerosis. *Circulation Research*.

[55] Jefferies C. A., Doyle S., Brunner C. (2003). Bruton's Tyrosine Kinase Is a Toll/Interleukin-1 Receptor Domain-binding Protein That Participates in Nuclear Factor *κ*B Activation by Toll-like Receptor 4. *Journal of Biological Chemistry*.

[56] Huang W., Morales J. L., Gazivoda V. P., August A. (2016). Nonreceptor tyrosine kinases ITK and BTK negatively regulate mast cell proinflammatory responses to lipopolysaccharide. *The Journal of Allergy and Clinical Immunology*.

[57] Sun M., Deng Z., Shi F. (2020). Rebamipide-loaded chitosan nanoparticles accelerate prostatic wound healing by inhibiting M1 macrophage-mediated inflammation via the NF-*κ*B signaling pathway. *Biomaterials Science*.

[58] He C., Jiang S., Yao H. (2018). Endoplasmic reticulum stress mediates inflammatory response triggered by ultra-small superparamagnetic iron oxide nanoparticles in hepatocytes. *Nanotoxicology*.

[59] Shenderov K., Riteau N., Yip R. (2014). Cutting edge: endoplasmic reticulum stress licenses macrophages to produce mature IL-1*β* in response to TLR4 stimulation through a caspase-8- and TRIF-dependent pathway. *Journal of Immunology*.

[60] Veith C., Neghabian D., Luitel H. (2020). FHL-1 is not involved in pressure overload-induced maladaptive right ventricular remodeling and dysfunction. *Basic Research in Cardiology*.

[61] Moon E. H., Kim Y. H., Vu P. N. (2020). TMEM100 is a key factor for specification of lymphatic endothelial progenitors. *Angiogenesis*.

[62] Hamczyk M. R., Villa‐Bellosta R., Quesada V. (2019). Progerin accelerates atherosclerosis by inducing endoplasmic reticulum stress in vascular smooth muscle cells. *EMBO Molecular Medicine*.

[63] Di Pasquale E., Condorelli G. (2019). Endoplasmic reticulum stress at the crossroads of progeria and atherosclerosis. *EMBO Molecular Medicine*.

[64] Wang Y., Wang W., Wang N., Tall A. R., Tabas I. (2017). Mitochondrial Oxidative Stress Promotes Atherosclerosis and Neutrophil Extracellular Traps in Aged Mice. *Arteriosclerosis, Thrombosis, and Vascular Biology*.

[65] Li C., Li L., Yang M., Zeng L., Sun L. (2020). PACS-2: a key regulator of mitochondria-associated membranes (MAMs). *Pharmacological Research*.

[66] Margadant C. (2020). Positive and negative feedback mechanisms controlling tip/stalk cell identity during sprouting angiogenesis. *Angiogenesis*.

